# Tolerance of Perovskite Solar Cells under Proton and Electron Irradiation

**DOI:** 10.3390/ma15041393

**Published:** 2022-02-14

**Authors:** Pei Li, Hua Dong, Jinghui Lan, Yurong Bai, Chaohui He, Liya Ma, Yonghong Li, Jiaxin Liu

**Affiliations:** 1 School of Nuclear Science and Technology, Xi’an Jiaotong University, Xi’an 710049, China; lipei0916@xjtu.edu.cn (P.L.); donghuaxjtu@mail.xjtu.edu.cn (H.D.); baiyur@stu.xjtu.edu.cn (Y.B.); yonghongli@mail.xjtu.edu.cn (Y.L.); 3120103348@stu.xjtu.edu.cn (J.L.); 2 Beijing Research Institute of Near Space Vehicle System Engineering, Beijing 100076, China; alanjia@126.com; 3 Key Laboratory of Functional Materials and Devices for Special Environments, Chinese Academy of Sciences, Urumqi 830000, China; maliya@ms.xjb.ac.cn

**Keywords:** perovskite solar cells, radiation effect, proton and electron irradiation, particle transport simulation

## Abstract

In this work, radiation experiments and simulations were carried out on perovskite solar cells (PSCs). The experimental results show that the PSCs in this work were robust to proton irradiation but more sensitive to electron irradiation, which is different from the results of previous studies. Simulations based on the Monte Carlo method show that the energy loss at the interface was much higher than that in the material bulk, and the interface was more sensitive to electron incidents.

## 1. Introduction

Solar technology is vitally important for space power applications, especially the Moon, Mars and other deep space exploration missions. Companies such as SpaceX are pressing ahead with the “Starlink” project, which is composed of 12,000 communication satellites, increasing the demand of low-cost solar cells. Currently, the majority of space solar technology is made with III-V compound solar cells such as the GaAs solar battery, which offers excellent efficiency and high tolerance to the space radiation environment [1,2,3]. However, the high cost and poor mechanical strength of the pure GaAs crystal make it difficult to use for large-scale space explorations [4,5]. In the past 10 years, the use of perovskite materials has led to rapid advances in the efficiency of solar cells. Tong et al. reported that a mixed tin–lead organic–inorganic material has a low bandgap, long charge-carrier lifetime and efficiencies of around 25%, which, to our best knowledge, is the highest reported efficiency to date [6,7]. PSCs have become one of the most efficient and low-cost photovoltaic technologies thanks to the development of fabrication protocols, chemical compositions and phase stabilization methods [8,9,10]. 

More importantly, the great advantages of high specific power (W/g), flexibility and cost-effectiveness make PSCs a strong contender for space power applications in extreme environments [11,12,13]. The lifetime of PSCs is known to be limited by instability issues while operating in an atmosphere that includes oxygen and moisture [14,15,16]. However, the space environment lacks water, and the atmosphere is extremely thin; thus, PSCs may survive longer in space than in the Earth’s atmosphere. However, charged particles and rays with high energy in the space environment directly affect the physical and chemical characteristics of materials, which causes the conversion efficiency of PSCs to deteriorate. Thus, the radiation environment and tolerance of PSCs need to be considered. 

Some studies have focused on the radiation effect of PSCs. The first report involving radiation effects on PSCs was presented in 2016 by Felix Lang et al.; this work showed that organic–inorganic perovskites exhibit radiation hardness and withstand proton doses that exceed the damage threshold of crystalline silicon by almost 3 orders of magnitude [17]. Follow-up research also suggested that PSCs have strong resistance to proton irradiation with energies of 50 keV, 100 keV or 68 MeV. For electron irradiation, PSCs can sustain a fluence of 10^12^ cm^−2^ with the energy of 1 MeV electron [18,19,20,21,22]. Another study demonstrated that PSCs experience substantial degradation under gamma radiation; in particular, *J_sc_* and *PCE* decreased significantly with the increase in radiation dose [23]. 

In our work, 1 MeV electron and 3 MeV proton radiation experiments were performed on PSCs manufactured by Xi’an Jiaotong University, and current density–voltage (J–V) curves were measured before and after radiation. Furthermore, Monte Carlo simulations were used to analyze the process of particle transport in PSCs. Experimental and simulation results all suggest that the PSCs in this work are more sensitive to electron irradiation and robust to proton irradiation.

## 2. Experiments

### 2.1. Materials and Experimental Details

**Figure 1:**
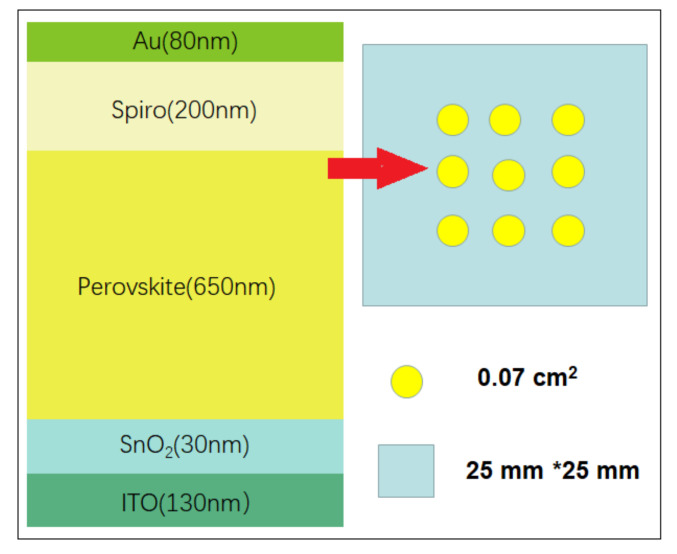
Cell structures of PSCs manufactured by Xi’an Jiaotong University.

The cell structures of PSCs in this work were designed and manufactured by Xi’an Jiaotong University. As illustrated in Figure 1, the order of layers is ITO (130 nm)/SnO_2_ (30 nm)/FA_0_._95_Cs_0_._05_PbI_3_ (650 nm)/Spiro (200 nm)/Au (80 nm). The quartz substrate of the cell was coated with indium tin oxide (ITO) to make the surface transparent and highly conductive, and the thickness of ITO was 130 nm. Metal oxides such as SnO_2_ are thermally stable and widely employed as electron transport materials (ETMs), and the thickness and composition were formed by tuning the chemical bath deposition of SnO_2_. For the perovskite absorber, formamidinium (FA) and Cs were chosen as cations with I and Br as halides, as they tend to exhibit high efficiency and robust stability. The organic material Spiro-OMeTAD was deposited as the hole transport materials (HTMs), which must cap the perovskite layer at low temperature to avoid the degradation of PSCs at high temperatures (>80 °C). The cell size was 0.07 cm^2^, and every 9 samples were packed into a quartz substrate with an area of 2.5 cm × 2.5 cm.

**Figure 2:**
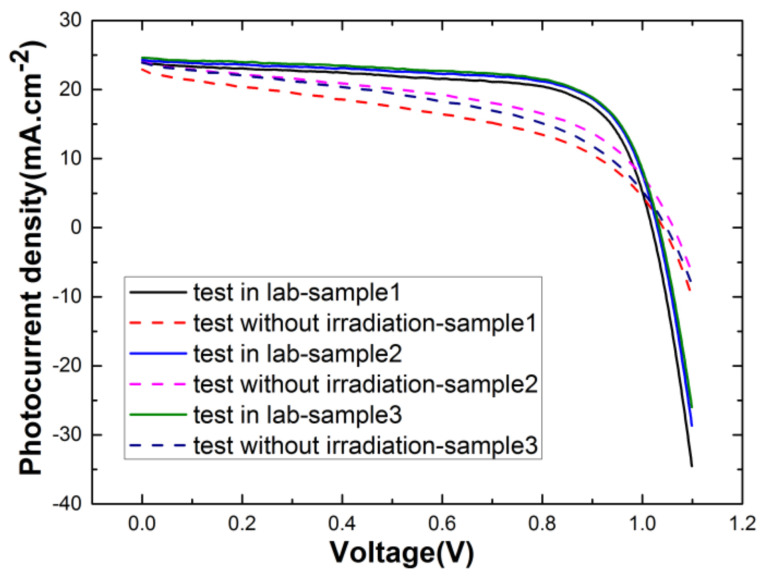
J–V curves of control groups were tested in the lab and natural environment without irradiation.

In order to exclude the effects of natural degradation, the response of control groups is illustrated in Figure 2, which shows a relatively small change in current density in the natural environment compared to that in the lab. For each of the irradiation and test runs, the sample quantities were set to 3. The electron irradiation experiments were conducted at the Key Laboratory of Functional Materials and Devices for Special Environments. The energy of the electron beam was continuously adjusted from 1.0 to 2.0 MeV, and the samples directly faced the electron beam to ensure that samples under irradiation received the same dose. The energy of the electron irradiation in this experiment was set to 1 MeV with flux stabilized at 10^11^ cm^−2^ s^−1^, and the fluence ranged from 10^14^ to 10^16^ cm^−2^. We also conducted a proton irradiation experiment with energy of 3 MeV in a vacuum environment using the electrostatic accelerator at Peking University. The flux of proton irradiation can be set to vary from 1.0 × 10^8^ to 6.5 × 10^8^ cm^−2^ s^−1^. In this work, the samples were irradiated at fluences of 1.0 × 10^11^, 1.0 × 10^12^ and 1.0 × 10^13^ cm^−2^, and the flux was stabilized at 10^11^ cm^−2^ s^−1^.

Establishing the current density–voltage (J–V) curve is an important method to represent the characteristics of solar cells. In this work, bias voltages were set to positive and negative polarity and then changed gradually under AM1.5 standard sunlight to obtain the corresponding J–V characteristics. Some other important parameters for evaluating solar cells include open-circuit voltage (*V_oc_*), short-circuit current (*J_sc_*), fill factor (*FF*) and power conversion efficiency (*PCE*), which were tested as well. The definitions of these parameters and the relationship between them are described below. When the output currents and applied bias voltages of solar cells are zero, they correspond to *V_oc_* and *J_sc_*, respectively. The parameter *FF* can be defined as in Formula (1), and *J_mp_* and *V_mp_* correspond to the current and voltage of the maximum output power point (*P_max_*) on the J–V curve, respectively. The higher the *FF*, the greater the rate of photon utilization. The parameter *PCE* characterizes the efficiency of solar cells in converting light energy into electrical energy, and it can be comprehensively defined by three parameters, as in Formula (2): *FF*, *J_sc_* and *V_oc_*.

(1) $$FF=\frac{J_{mp}\times V_{mp}}{J_{sc}\times V_{oc}}$$

(2) $$PCE\%=\frac{P_{max}}{P_{\mathrm{in}}}=\frac{FF\times J_{sc}\times V_{oc}}{P_{\mathrm{in}}}\times 100\%$$

### 2.2. J–V Analysis

The J–V curve can be directly used to characterize the electrical performance of solar cells. Figure 2 shows the J–V curves of PSCs with different electron irradiation doses under AM1.5 sun illumination. The parameters *J_sc_*, *V_oc_*, *FF* and *PCE*% under electron and proton radiation are all listed in Table 1. For electron irradiation, the parameters all gradually degenerated with a fluence ranging from 1.0 × 10^11^ to 1.0 × 10^13^ cm^−2^, while there was nearly no change in parameters with increasing proton fluence.

**Figure 3:**
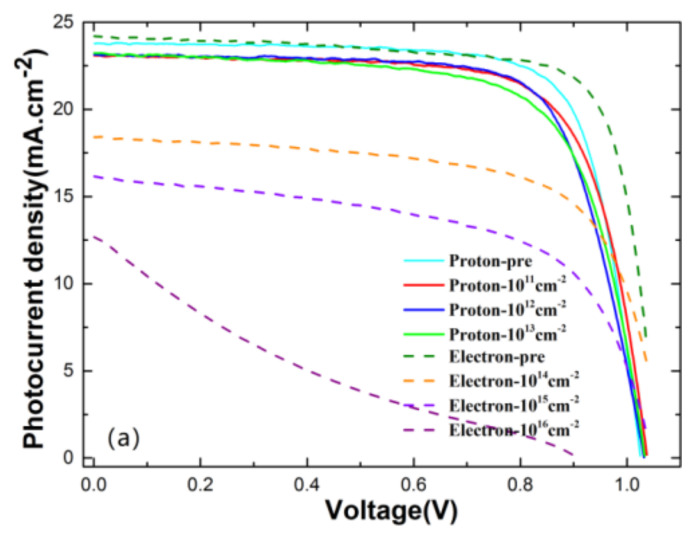

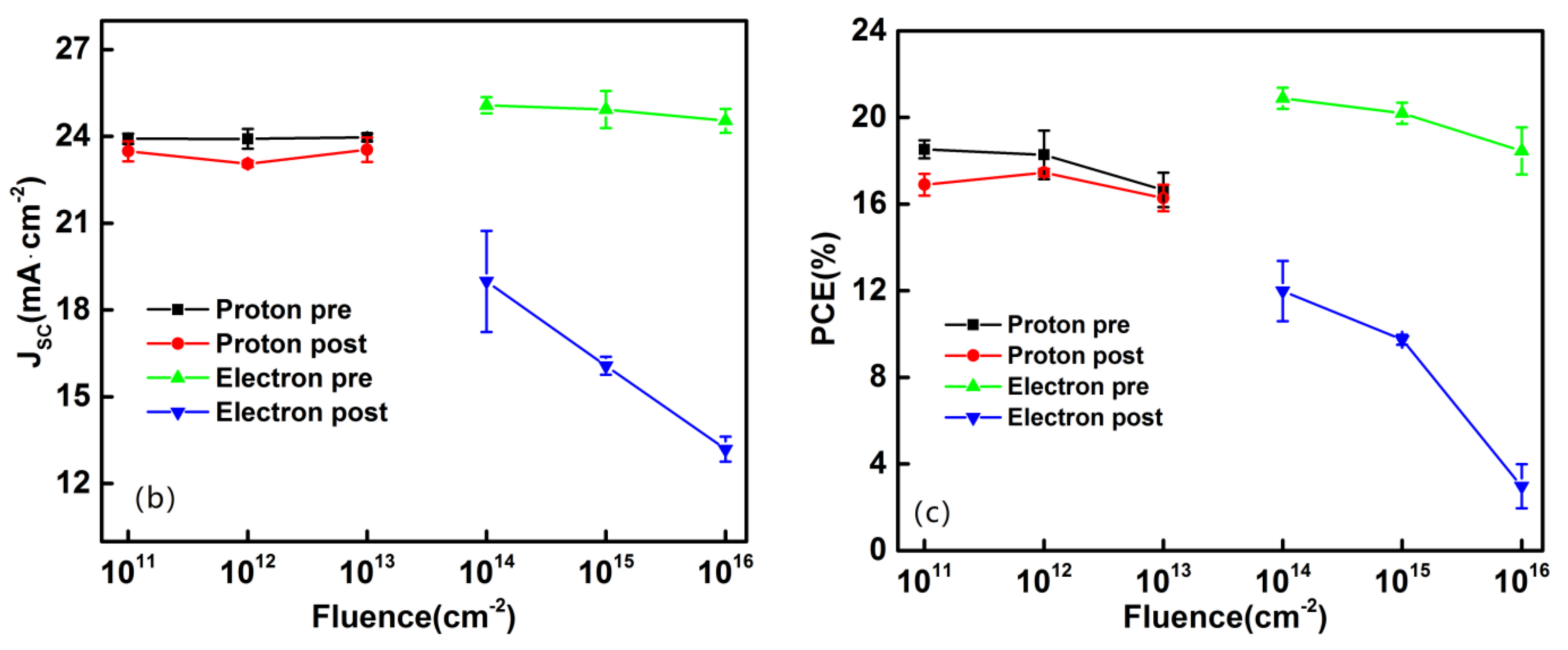
(**a**) Photocurrent density, (**b**) *J_sc_* and (**c**) *PCE*% under electron and proton irradiation with different fluences.

As illustrated in Figure 3a, there was nearly no degradation of the photocurrent density induced by proton radiation, while the degradation induced by electron radiation was more severe than that under proton radiation, especially the photocurrent density with a fluence of 10^16^ cm^−2^, which led to a typical device failure. As is clearly shown in Figure 3b, the short-circuit currents of PSCs experienced a significant decrease with accumulating electron fluence, while for proton radiation, there were nearly no significant changes observed in *J_sc_* curves. The parameter *PCE*% characterizes the photoelectric conversion efficiency of solar cells. As we can see in Figure 3c, PSCs exhibited a relatively stable *PCE* of 16~20% under proton radiation, but it dropped significantly under electron radiation. With that being stated, it is reasonable to conclude that the PSCs tested in this work are more sensitive to electron irradiation than proton radiation. Previous studies also demonstrated that PSCs can survive against accumulated proton fluence of up to 1.0 × 10^14^ cm^−2^ with energies of 50 keV, 100 keV and 68 MeV. In sum, despite the different energies and sample types, the experimental results show similar trends that suggest that PSCs have strong tolerance to proton irradiation. However, previous research with electron radiation showed that PSCs can survive against accumulated dose levels of up to 1.0 × 10^16^ cm^−2^ with the same energy of 1 MeV, which is different from the response to electron radiation in our work [8].

## 3. Particle Transport Simulation

The degradation of solar cells under proton radiation is known to be primarily induced by a cluster of interstitial lattice atoms and an equivalent number of vacancies. In contrast to proton radiation, electron damage to the lattice is characterized by point defects that are considerably more uniform throughout the lattice than those that occur with proton damage [24,25]. Although the mechanisms of electron and proton damage are different, all of these damage centers can alter the minority-carrier lifetime, and the greater the number of centers, the lower the lifetime of minority carriers. At present, the physical mechanism of interaction between particles and PSC material remains unclear. Some studies have shown that the F centers play a very important role in the performance of perovskite materials, but fundamental parameters such as formation energy and energy level need further investigation [26]. Another study showed that proton irradiation improves the performance of PSCs, and the point defects induced by proton radiation due to the displacements of atoms in the inorganic Pb-I framework act as unintentional doping sources and partially compensate for deep traps originating from the photodegradation of methylammonium molecules [27]. 

**Figure 4:**
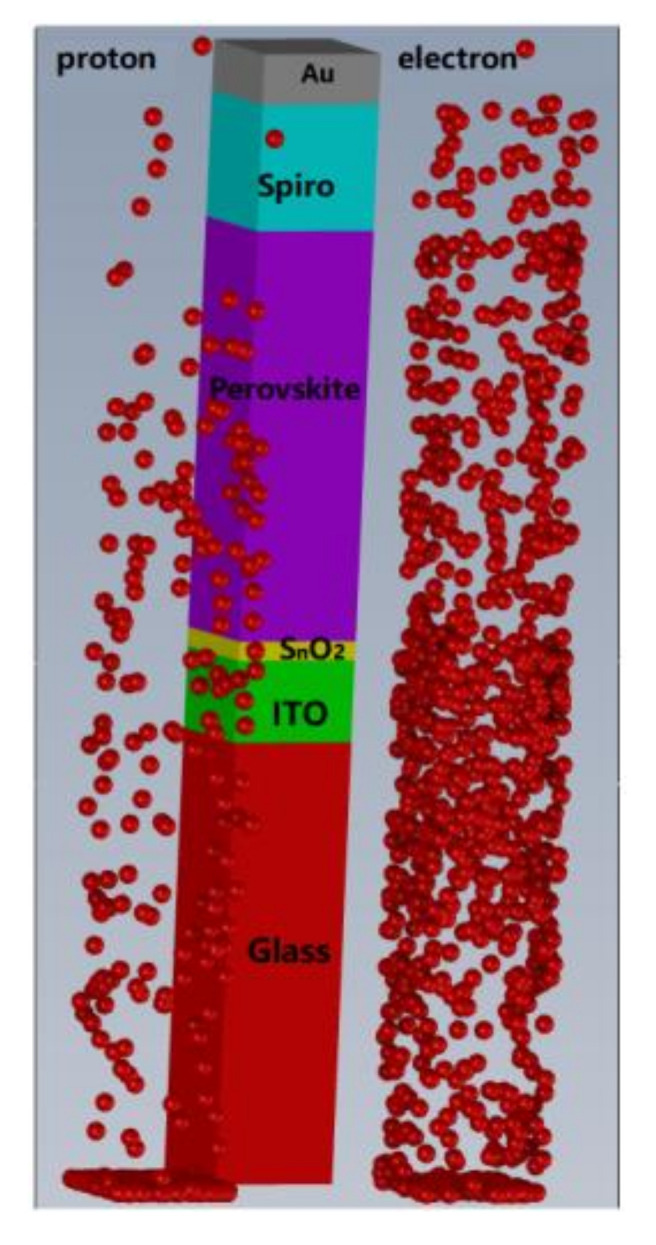
A 3D view showing 1 MeV electrons and 3 MeV protons reacting with the perovskite solar cell.

In this work, the Monte Carlo software Geant4 (Geant4.10.06, European Organization for Nuclear Research, Geneva, Switzerland) was used to simulate particle transport in PSCs and further analyze the sensitivity to electron radiation and the tolerance to proton radiation. Geant4 simulation requires several steps, including detector modeling, physical process, particle source and data acquisition. The detector model is shown in Figure 4, which contains six material layers, namely, Glass (700 nm), ITO (130 nm), SnO_2_ (30 nm), FACsPbI (650 nm), Spiro (200 nm) and Au (80 nm), and the total thickness of the device is 1790 nm. The physical models of electron and proton irradiation were obtained from a standard library and are written in the file PhyListEmStandard.cc. A surface source with an area of 200 nm × 200 nm was used to simulate particle transport in perovskite solar cells, and the initial direction and position are defined in the file PrimaryGeneratorAction.cc. The various stages of particle transport, energy loss calculation and data collection were all controlled by the files SteppingAction.cc, RunAction.cc, etc., [28,29].

As illustrated in Figure 4, 1000 electrons with energy of 1 MeV and 1000 protons with energy of 3 MeV were set up to enter the devices separately. The range of 3 MeV protons calculated by Geant4 is 73.5 μm, which is far greater than the total thickness of the PSCs in this work (1790 nm). Most protons had to penetrate the PSCs and cause uniform damage without causing a significant collision event, while the movement track of electrons was very tortuous, and nearly all electrons stopped within the cell layers.

To further analyze the transport of high-energy particles, 1.0 × 10^10^ electrons and 1.0 × 10^7^ protons were separately set up to enter the devices, and the number of incidents was based on the experimental fluence. 

**Figure 5:**
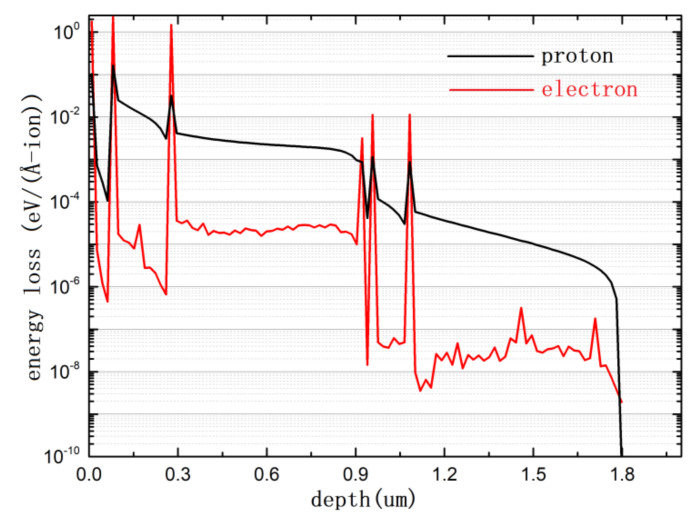
Energy loss varies with the depth of material layers.

As illustrated in Figure 5, the energy losses of electrons and protons were calculated and found to vary in their distribution with the depth of material layers. As expected, the energy loss of protons was much higher than that of electrons, and the curve of protons was much smoother than that of electrons because of their large stopping cross-section and the greater mass of a proton compared to an electron (>2000 times). An interesting observation is that there were several peaks of energy loss at the interface of different materials. In addition, the energy loss of electrons was about 1 order of magnitude larger than that of protons at the material interface and 2 orders of magnitude smaller than that of protons in the bulk. This indicates that the interface was more sensitive to particle incidents, especially those involving electrons. This may be the reason that the PSCs in this work appeared to be more sensitive to electron irradiation and robust to proton irradiation for the same cell structure.

## 4. Conclusions

At present, perovskite solar cells (PSCs) are one of the most efficient and low-cost solution-processable photovoltaic technologies. The need for low-cost solar cells and a working environment without moisture make PSCs a strong contender for space applications. Several studies have demonstrated that PSCs have high stability under proton radiation regardless of the energy (varying from keV to MeV), and the resistance to electron radiation reached a fluence of 1.0 × 10^16^ cm^−2^. 

In our work, the experimental results show similar trends suggesting that PSCs have strong tolerance to proton irradiation, but their characteristics exhibit significant degradation with accumulating fluence, which is different from the results of previous studies. In addition, the simulation results show that the energy loss of protons was much higher than that of electrons in the bulk of PSCs, as expected. However, it was exactly the opposite at interfaces between different materials, which were more sensitive to electron incidents, and the energy loss was about 1 order of magnitude larger than that of proton incidents at the interface. In conclusion, the resistance to proton radiation was confirmed once again, while the responses to electron radiation varied from cell to cell. 

## Figures and Tables

**Table 1:** Results of electron and proton irradiation experiment.

Parameter		*J_sc_*/mA·cm^−2^		*V_oc_*/V		*FF*		*PCE*%	
Structure and Fluence		Pre	Post	Pre	Post	Pre	Post	Pre	Post
Electron	**1 × 10^14^**	25.05	20.95	1.06	1.00	0.771	0.501	20.53	10.50
	**1 × 10^15^**	25.43	16.32	1.06	1.06	0.757	0.556	20.30	9.62
	**1 × 10^16^**	24.93	13.49	1.05	1.00	0.743	0.301	19.45	4.05
Proton	**1 × 10^11^**	23.78	23.09	1.03	1.04	0.758	0.725	18.49	17.39
	**1 × 10^12^**	24.12	23.13	1.06	1.03	0.748	0.726	19.18	17.34
	**1 × 10^13^**	23.84	23.24	0.98	1.03	0.674	0.695	15.80	16.71

## Data Availability

Data sharing is not applicable to this article.
